# Elemental mass spectrometry to study metallo-transcriptomic changes during the in vitro degeneration of the retinal pigment epithelium

**DOI:** 10.1007/s00216-023-04880-8

**Published:** 2023-07-29

**Authors:** Ana Álvarez-Barrios, Lydia Álvarez, Rosario Pereiro, Héctor González-Iglesias

**Affiliations:** 1 Fundación de Investigación Oftalmológica, Avda. Dres. Fernández-Vega. 34, 33012 Oviedo, Spain; 2 Department of Physical and Analytical Chemistry, University of Oviedo, Julián Clavería, 8, 33006 Oviedo, Spain; 3 Instituto Oftalmológico Fernández-Vega, Avda. Dres. Fernández-Vega, 34, 33012 Oviedo, Spain; 4 Instituto de Productos Lácteos de Asturias, Consejo Superior de Investigaciones Científicas (IPLA-CSIC), Villaviciosa, Spain

**Keywords:** ICP-MS, Trace elements, Transcriptomics, Retinal pigment epithelial cells, Cell culture, Homeostasis

## Abstract

Trace elements play crucial roles in cellular biology. Their improper homeostasis may contribute to the progress of eye diseases, exacerbated during ageing. The retinal pigment epithelium (RPE) is progressively deteriorated during age-related neurodegeneration and metal homeostasis may be compromised. In this study, elemental mass spectrometry (MS) was combined with cellular and molecular biology techniques to identify changes in trace elements during the in vitro degeneration of human RPE cells. Cells were collected at 21, 91, and 133 days and processed for RNA sequencing; Ca, Na, P, Mg, and Cu quantification by flow injection analysis and inductively coupled plasma–MS; and protein analysis by immunocytochemistry. Four-month-old RPE cultures showed depigmentation, impaired barrier function, and antioxidant protection, manifesting signs of epithelial-to-mesenchymal transition. Na and P significantly increased in the cytosol of degenerated RPE cells (from 15 ± 20 to 13495 ± 638 ng·µg^−1^ and from 30.6 ± 9.5 to 116.8 ± 16.8 ng·µg^−1^, respectively). Mg decreased in both the cytosol and insoluble fraction of cells (from 2.83 ± 0.40 to 1.58 ± 0.56 ng·µg^−1^ and from 247.57 ± 11.06 to 30 ± 8 ng·g^−1^, respectively), while P and Cu decreased in the insoluble fraction after 133 days in culture (from 9471 ± 1249 to 4555 ± 985 ng·µg^−1^ and from 2251 ± 79 to 1054 ± 235 ng·g^−1^, respectively), along with changes in metal-dependent antioxidant enzymes and Cu transporters. This RPE model reflected metal homeostatic changes, providing additional perspectives on effects of metal regulation during ageing.

**Graphical Abstract:**

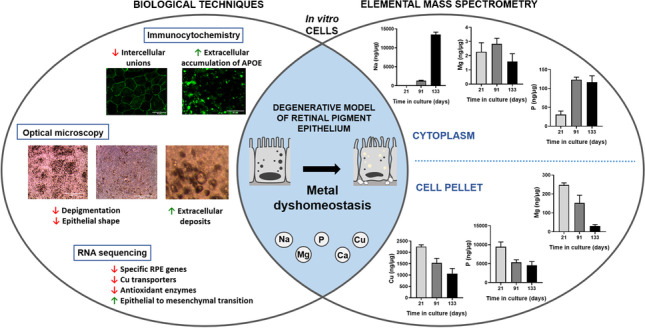

**Supplementary information:**

The online version contains supplementary material available at 10.1007/s00216-023-04880-8.

## Introduction

Trace elements and minerals are mandatory in cellular physiology, requiring an adequate homeostatic system to control their levels considering their tight biological window [1]. During ageing, elemental homeostasis is altered, contributing to cell toxicity, free radical production, inflammation, protein misfolding, abnormal deposition, and cell apoptosis [2]. Age is a risk factor of many eye diseases, mainly those characterized by the progressive deterioration of the retina, as it happens during age-related macular degeneration (AMD), one of the main causes of irreversible blindness [3]. Progression of this macular disease primarily affects the structure and function of the retinal pigment epithelial (RPE) cells, a monolayer of hexagonal cells responsible for the nourishment of the neuronal retina [4]. Degeneration of the RPE during AMD triggers the formation of extracellular sub-RPE deposits known as drusen and containing lipids, minerals, and aberrant aggregation of proteins [5]. In this vein, there is a current need for understanding the role of essential elements, including metals, in the function and disease of the visual system.

Elemental mass spectrometry (MS) is a highly sensitive, accurate, and versatile technique with a huge perspective in elemental bioanalysis. The inductively coupled plasma–MS (ICP-MS) has been widely used to study metal homeostasis and their role in biomedical applications, alone or after coupling with separation techniques [6, 7]. The multielement analysis ability for the determination of trace elements in biofluids, cells, and tissues, providing high dynamic range and low sample consumption, offers great advantages for ICP-MS application in biological sciences. Within the eye, a huge number of publications using ICP-MS emerged in last years, contributing to the understanding of the role of metals in eye health and disease [8, 9]. However, ICP-MS itself lacks additional information about the relationship of essential elements with genes and proteins, and its combination with other bioanalytical techniques including transcriptomics, proteomics, or classical cell biology may contribute to a better understanding of the role of metals during AMD onset and progressive degeneration of the RPE.

The development of in vitro models of RPE cells able to reproduce the formation of extracellular deposits with characteristics of drusen provides many advantages for studying trace elements homeostasis [10]. In a previous study, we established a model of matured RPE cells cultured in a 2D monolayer with features of early AMD [11]. Their 2-month follow-up provided evidence of normal RPE process development, early deposition of sub-RPE material, and consequent changes in Zn levels suggesting altered Zn homeostasis during RPE maturation and early abnormal protein accumulation. However, there is a lack of understanding of the role and changes of trace elements on degenerated RPE cells, being mandatory in the study of long-term cultured RPE cells during their progressive atrophy and resulting formation of extracellular deposits of pathogenic proteins. Hence, a multidisciplinary approach, combining biological techniques with ICP-MS, can give a wider insight into the state of cells and help to depict the role of trace elements during RPE deterioration. In addition, working with cell cultures has specific limitations from an analytical point of view, mainly the heterogeneity of cells due to the intrinsic variability of biological processes and the low intracellular concentration of trace metals. Thereby, sample introduction systems using low sample volume for ICP-MS, such as flow injection analysis (FIA) used in this work, provide a versatile option to reduce sample consumption and analyze analytes of limited concentration and volume like cell extracts.

In this spirit, the aim of this work was to implement an analytical platform based on FIA-ICP-MS to improve the knowledge in the relationship between metals, ageing, and RPE degeneration using, as a case model, the identification of potential age-related changes relevant to AMD disease. For such purposes, the transcriptome, specific proteome, and metal (Ca, Na, Mg, and Cu) and non-metal (P) levels of an in vitro RPE cell culture model were studied during its progressive degeneration followed up to 4 months.

## Material and methods

### RPE cell culture conditions

Primary human fetal RPE cells (ScienCell, Cat. No. 6540) were used at passage three (P3) and seeded in 12-well Transwell® plates (Corning Inc., Cat. No. CLS3460, density of 142,900 cells·cm^−2^) coated with 2% v/v Geltrex® matrix (Thermo Fisher Scientific; Cat. No. A1413202) following previous procedures [10, 11]. Culture Miller media was renewed 2–3 days a week and cells were monitored for 4 months using the optical microscope Leica DM II LED (Leica Microsystems) and the Millicell ERS-2 Voltohmmeter (Merck Millipore, ref. MERS00002) to measure transepithelial electrical resistance (TEER). Metal levels, gene expression, and protein localization were independently determined in RPE cells cultured on separate inserts (nine independent inserts for each analytical approach).

### Immunoassays

Cells at 21, 91, and 133 days in culture were fixed with 4% *v/v* paraformaldehyde and stored at 4 °C in PBS until immunostaining of zonula occludens-1 (ZO-1), claudin-19 (CLDN19), bestrophin-1 (BEST1), and apolipoprotein E (APOE) proteins [11]. Briefly, permeabilized and PBS-washed cells were incubated for 1.5–2 h in a solution of 1% *v/v* BSA (Sigma-Aldrich, ref. A9418-10G) and 10% *v/v* serum (from goat (Vector Laboratories, ref. S-1000) in the case of ZO-1, CLDN19, and BEST1; and from donkey (Jackson ImmunoResearch Europe Ltd, ref. 017–000-121) in the case of APOE) in PBS, and later incubated overnight at 4 °C with the primary antibodies 1:100 *v/v* anti-ZO1 antibody (Thermo Fisher Sci., ref. 617,300), 1:100 *v/v* anti-CLDN19 antibody (Novus, ref. H00149461-M02), 1:50 *v/v* anti-BEST1 antibody (Sigma-Aldrich, ref. MAB5466), and 1:100 *v/v* anti-APOE (Sigma-Aldrich, ref. AB947), in PBS (0.1% *v/v* BSA and 10% *v/v* host serum). After incubating with 1:500 *v/v* secondary antibodies AF 488 or AF 594, samples were mounted on glass slides using DAKO (Agilent Technologies, ref. S302380) for its visualization by fluorescence microscopy (Leica DMI6000 B, Leica Microsystems).

### RNA isolation, sequencing, and statistical analysis

RNA from culture cells at 21, 91, and 133 days (3 biological replicates per time) was extracted using RNeasy Mini Kit (Qiagen) following the manufacturer’s procedures. The RNA quality control, library construction, RNA sequencing, and quantitative analysis were performed in BGI Genomics (Beijing Genomics Institute, Shenzhen, China), using the BGISEQ-500 platform, as previously published [11]. The bioinformatic platform Dr. Tom (http://biosys.bgi.com) was used for statistics, where pairwise comparisons were made between age groups. The inter-group differential expression analysis was conducted using DESeq2 [12]. Significant DEGs were identified with *Q*-values (adjusted *P*-value) ≤ 0.05. Enrichment analyses of the biological process Gene Ontology (GO) terms for DEGs between age groups were obtained (*Q*-value ≤ 0.05 significantly enriched in DEGs).

### Elemental mass spectrometry for multielemental analysis of RPE cells

The Agilent 7900 ICP-MS instrument was used for the multielemental quantification of Ca, Na, Mg, P, and Cu levels in both soluble and insoluble fractions. A flow injection analysis (FIA) system consisting of a Rheodyne™ six-port valve fitted with a 5μL sample loop was used for sample introduction. Samples were quantified in triplicates by external calibration using Ga as the internal standard, considering is not normally present in appreciate quantities in RPE cells and properly corrects possible suppression of the ionization signal and instrumental drift, and 1% *w/w* HNO_3_ was used as eluent throughout the FIA-ICP-MS system. Specific instrumental parameters are summarized in Table 1 and analytical parameters for FIA-ICP-MS analysis are shown in Table S1 of Supplementary Information. RPE cells at 21, 91, and 133 days in culture were washed with Ca- and Mg-depleted Dulbecco’s phosphate-buffered saline (DPBS; ScienCell, Cat. No. 0303), cut from the plates with the polyester membrane, and transferred to an Eppendorf tube containing 200 µL Tris–HCl buffer. Cells were lysed by ultra-sonication on an ice bath (three cycles of 30 s at 10 kHz) and centrifuged at 16,000 g for 20 min to obtain the water-soluble cell fraction (containing cytoplasm) and an insoluble fraction (containing cell nuclei and plasma membranes), following a previous protocol [11, 13]. The insoluble fraction (pellet) was digested with 50 µL of HNO_3_ (68% *w/w*, TraceMetal™ Grade, Fisher Chemical) assisted by the use of an ultrasonic bath (Fisher Scientific, UK) for 30 min. Protein content was determined in the water-soluble fraction using the commercial QuantiPro™ BCA Assay Kit (Sigma-Aldrich, Cat No. QPBCA) for normalization. Multielemental content of Ca, Na, Mg, P, and Cu was also determined by FIA-ICP-MS in the culture media, without observing statistically significant differences during the follow-up (see Table S2 of Supplementary Information). Prism 9 software (GraphPad) was used for statistical analysis using a two-way ANOVA test.

**Table 1:** ICP-MS operating conditions for the analysis of trace elements in RPE cells using FIA system

Plasma generation	RF power	1550 W
	Plasma flow	15 L min^−1^
	Nebulizer gas flow	1.07 L min^−1^
	Auxiliary gas flow	0.90 L min^−1^
Collision cell	He flow	4.5 mL min^−1^
	Octopole voltage	− 18 V
	Quadrupole voltage	− 13 V
Data acquisition	Acquisition mode	Time-resolved analysis
	Monitored isotopes	^23^Na, ^24^ Mg, ^31^P, ^44^Ca, ^63,65^Cu, ^69,71^ Ga
	Points per peak	10
	Acquisition time per point	0.31 s
Sample introduction (flow injection analysis)	Sample flow rate	0.32 mL min^−1^
	Injection volume	5 μL
	Replicates	3

## Results and discussion

In this work, the combination of ICP-MS with RNA sequencing and immunohistochemistry has been explored as an analytical platform to identify metallo-transcriptomic changes associated with the in vitro degeneration of the RPE. The RPE, a component of the blood-retinal-barrier, is a monolayer of polarized hexagonal cells contributing to retinal homeostasis and cellular adhesion [4]. During ageing, the RPE undergoes structural changes including loss of melanin, oxidative stress increase, progressive accumulation of extracellular deposits, and degeneration, as occurs during AMD onset [5]. In this vein, a simplified RPE model able to reproduce the formation of extracellular deposits with characteristics of drusen has been established following the previous work of Pilgrim et al. [10], in which a physical barrier was imposed against the movement of RPE-secreted material.

In a previous study, the 2-month follow-up of RPE cells provided a picture of the RPE during its maturation and the early accumulation of extracellular deposits. Particularly, RPE cells showed normal epithelial processes, presence of neuroepithelial proteins, and initial deposition of sub-RPE material, concurring with an altered Zn homeostasis, exacerbated by changes in cytosolic Zn-binding proteins and Zn transporters. In the current study, RPE cell cultures were followed during their in vitro degeneration, with a maximum culture time of 133 days (i.e., up to 4 months). These cells still produce abnormal extracellular deposits, but in addition, progressive atrophy of cultured RPE cells occurred, which may be considered an in vitro long-term degeneration RPE model, probably mimicking advanced stages of AMD.

### Follow-up of specific markers of RPE, tight junction proteins, and barrier function

**Fig. 1:**
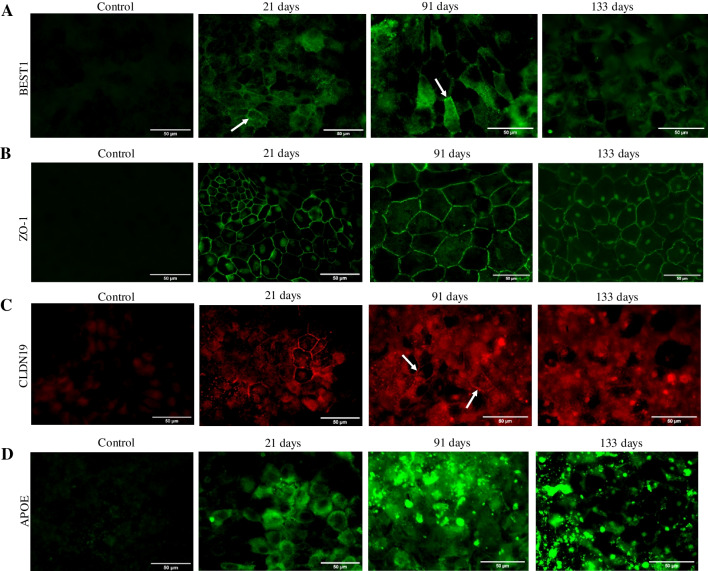
Protein immunolocalization of BEST1, ZO-1, CLDN19, and APOE in RPE cells at 21, 91, and 133 days in culture. Control refers to RPE cells (21 days) not exposed to the primary antibody. **A** BEST1 protein was detected in the cell membranes of some RPE cells after 91 days (arrow), but not after 133 days in culture. **B** ZO-1 is detected in the cell membranes of RPE cells after 91 and 133 days in culture, although at 133 days some cell detachment is observed. **C** Some CLDN19 signals were observed in the cell membranes of 91-day-old RPE cells (arrows), while no CLDN19 was detected after 133 days. **D** APOE is detected in the cytoplasm of RPE cells at 91 and 133 days in culture, together with intense extracellular signals possibly linked to deposit formation

First, we confirmed that RPE cells expressed makers of the native epithelium [14], including the genes of the visual cycle RPE-retinal G-coupled receptor (*RGR*), lecithin retinol acetyltransferase (*LRAT*), bestrophin 1 (*BEST1*), retinaldehyde binding protein 1 (*RLBP1*), and retinal pigment epithelium-specific 65 kDa protein (*RPE65*), which expression decreased from 91 to 133 days in culture, according to its progressive degeneration (Table 2). At the protein level, immunostaining of transmembrane protein BEST1 revealed its presence in the cell membrane at 21 days of culture, also detected in some cell membranes at 91 days but faded after 133 days (Fig. 1A), as occurs during Best disease [15] reflecting, along with reduced expression of other visual cycle and melanin biosynthesis genes, the RPE degeneration or dedifferentiation.

**Table 2:** Gene expression of RPE markers in the cell cultures. Several of the analyzed RPE markers are significantly downregulated at the end of the experiment (133 days), with the exception of PAX6 and RDH5. ZO-1 coding gene, *TJP1*, is significantly downregulated at 91 and 133 days in comparison to 21 days in culture. Gene expression of *CLDN19* significantly decreased after 91 and 133 days in comparison to 21 days in culture. Differential gene expression analysis was carried out following the DESeq2 method (ns: *q*-value > 0.05; *: *q*-value < 0.05; **: *q*-value < 0.01)

	Average read count			Fold change (significance)		
Gene	21 days	91 days	133 days	91 vs 21 days	133 vs 91 days	133 vs 21 days
*BEST1*	4485	5582	33	1.32 (ns)	0.01 (**)	0.01 (**)
*DCT*	5539	1103	43	0.21 (**)	0.04 (**)	0.01 (**)
*LRAT*	151	397	6	2.79 (**)	0.02 (**)	0.04 (**)
*MITF*	2141	1816	520	0.91 (**)	0.31 (ns)	0.28 (**)
*OTX2*	5733	3395	878	0.63 (**)	0.28 (**)	0.17 (**)
*PAX6*	913	1963	1270	2.32 (**)	0.68 (**)	1.59 (**)
*RDH5*	1026	985	646	1.02 (ns)	0.70 (ns)	0.72 (ns)
*RGR*	396	1839	6	4.92 (**)	0.00 (**)	0.02 (**)
*RLBP1*	672	987	10	1.56 (**)	0.01 (**)	0.02 (**)
*RPE65*	144	322	2	2.37 (**)	0.01 (**)	0.01 (**)
*SERPINF1*	90,542	43,036	4409	0.51 (**)	0.11 (*)	0.06 (*)
*TYR*	14,239	4373	149	0.33 (**)	0.04 (**)	0.01 (**)
*TYRP1*	82,041	23,698	1319	0.31 (**)	0.06 (**)	0.02 (**)
*TJP1*	3084	1962	1847	0.69 (**)	1.00 (ns)	0.68 (**)
*CLDN19*	2588	1804	46	0.02 (*)	0.03 (ns)	0.02 (*)
*APOE*	20,438	6449	1481	0.34 (**)	0.24 (**)	0.08 (**)

The intercellular tight junction proteins CLDN19 and ZO-1 (*TJP1* gene) after 21 days showed adequate levels and localization both at RNA and protein, while at the end of RPE degeneration, i.e., after 91 and 133 days in culture, also exhibited a decreased gene expression in comparison with 21 days. The ZO-1 protein stayed in the membranes of RPE cells throughout the 91–133-day period, even after cell junctions were lost at 133 days (Fig. 1B). Also, a loss of the protein signal CLDN19 was observed in the cell membranes at 91 and 133 days, when compared with 21 days in culture (Fig. 1C). The loss of CLDN19 at the end of 133 days in culture proves that intercellular unions may be lost, and the cellular barrier conformed by the RPE degenerates in vitro after long culturing times [16].

Cellular barrier function was monitored through TEER measurement, which values were normalized to the initial resistance at 4 days in culture. TEER increased until 21–35 days in culture, reaching a maximum of 180 ± 50%, and decreased afterwards to values below the initial ones after 112 days in culture (16 ± 8%) (Figure S1 of Supplementary Information), which indicates leaky tight junctions at the end of the degenerated RPE.

### Progressive degeneration of RPE cells in culture: extracellular deposit formation, loss of pigmentation, and enrichment of processes associated with degeneration

**Fig. 2:**
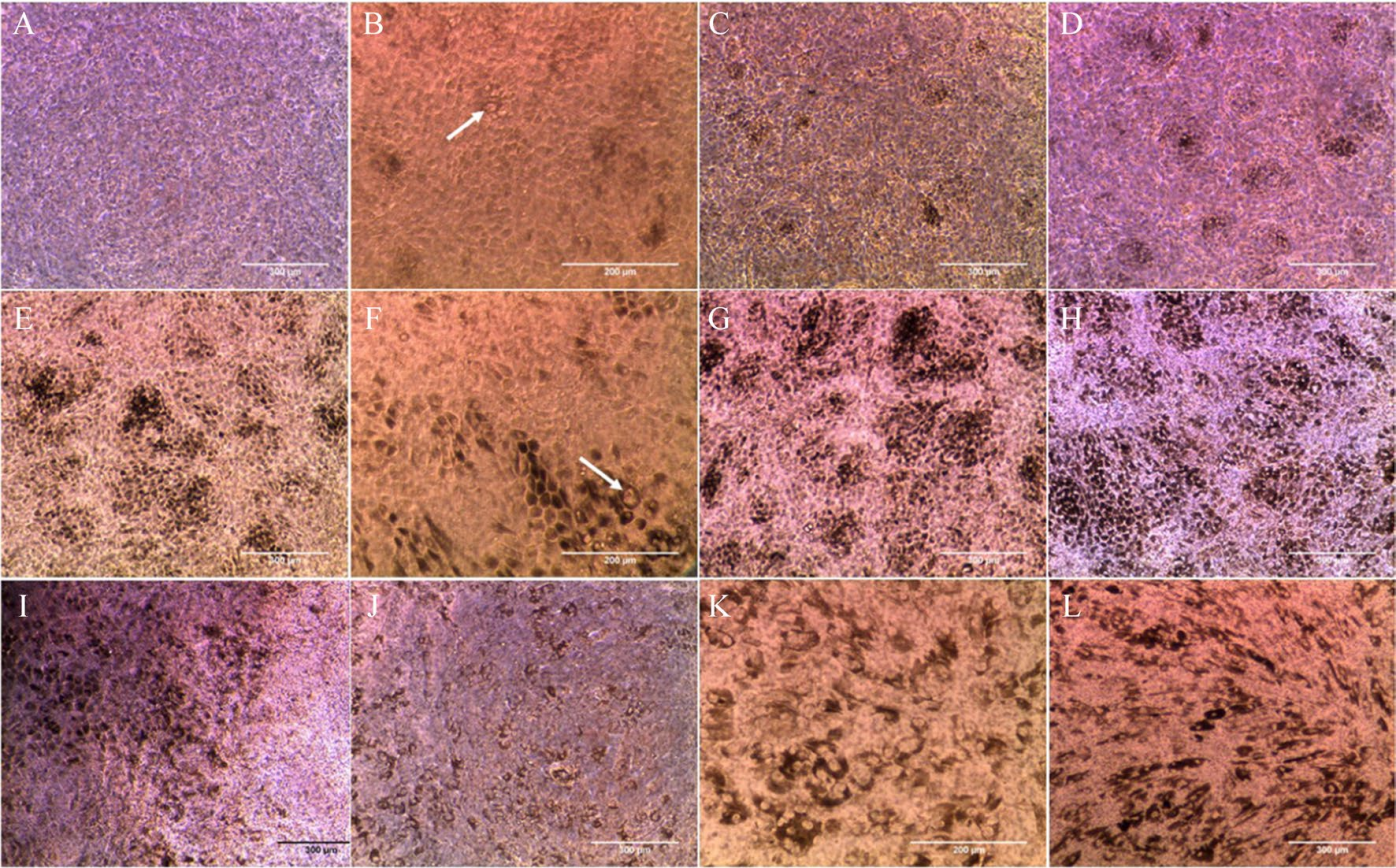
Optical microscopy images of the cell cultures at different time points during experiment: **A** 7 days; **B** 14 days; **C** 24 days; **D** 35 days; **E**–**F** 45 days; **G** 63 days; **H**–**I** 77 days; and **J**–**L** 115 days. Pigmentation of cells started at around 14 days in culture (**B**) and increased with time (**C**–**I**), mostly in the center (**H**) and borders of the inserts (**I**). Possible extracellular deposits (arrows) appeared after 14 days in culture (**B**), with high concentration in the insert borders (**F**). Cultures of 115 days showed signs of degeneration, such as less pigmentation (**J**) and atrophied cells (**K**–**L**)

Cellular morphology dramatically changed along culture (Fig. 2), with progressive loss of pigmentation and deformation of cell shape during RPE degeneration (Fig. 2J–L). Depigmentation of RPE cells was previously reported in porcine RPE cell cultures with time [17], and human eyes from donors aged between 60 and 90 years, which had 36% less melanin than their counterparts of 20–30 years [18]. Extracellular deposits were observed after 14 days in culture (Fig. 2B), concentrated near the walls of the cell inserts (Fig. 2F). Degeneration occurred at different rates throughout the cell culture, being the periphery and borders the most affected, while in most cases the central cells survived until the end of the experiment after 4 months in culture. The extracellular deposit formation observed during the in vitro degeneration of the RPE is one of the main clinical hallmarks of AMD [5]. During AMD, progressive accumulation of extracellular material induces a chronic inflammation (i.e., para-inflammation) and eventually may disrupt the exchange of nutrients and waste products between the RPE and blood vessels of the choriocapillaris, ultimately participating in the progressive degeneration of the RPE cells observed in AMD [19].

The capacity of cells in culture to form extracellular deposits characteristic of degenerating RPE was monitored by the RPE-secreted protein APOE follow-up, one of the main components found in drusen facilitating lipid accumulation [5]. *APOE* gene expression significantly decreased from 21 to 91 days in culture and from 91 to 133 days (Table 2). At the protein level, we found intense APOE protein signals in the extracellular space at 91 and, more prominently, at 133 days in culture (Fig. 1C), likely belonging to possible extracellular deposits. The detection of increased APOE deposition, which accumulation of molecules in the extracellular space forming abnormal deposits is a common phenomenon during ageing and neurodegeneration [10], confirmed that this simplified in vitro model of degenerated RPE cells over time showed some characteristics of AMD.

In addition, differentially expressed genes (DEGs) were identified and aligned to the Gene Ontology (GO) database for biological processes enrichment analysis. The top 20 most significantly enriched processes were selected and depicted as bubble charts in Figure S2 of Supplementary Information. The most significantly enriched biological processes between 21 and 133 days in culture can be attributed to (1) the maintenance of the RPE (GO term: “cell adhesion”); (2) basic functions of the mature RPE (GO term: “transmembrane transport”); (3) modification of biomolecules (GO terms: “phosphorylation,” “oxidation–reduction process,” and “post-translational protein modification”); (4) communication with other structures of the retina (GO terms: “axon guidance,” and “nervous system development”); and (5) apoptosis (GO terms: “negative regulation of apoptotic process,” “positive regulation of apoptotic process,” and “apoptotic process”). Alteration of processes like “cell migration,” “actin cytoskeleton organization,” “positive regulation of cell migration,” “extracellular matrix organization,” and “Wnt signalling pathway” could indicate some epithelial-to-mesenchymal transition (EMT) in the cell cultures [20].

### Epithelial-to-mesenchymal transition during RPE degeneration

Since several pathways that match biological events associated with EMT have been found to be altered during the RPE degeneration (Figure S2), we studied specific genes involved in EMT (Table 3). At the end of the 133rd day in culture, the genes E-cadherin (*CDH1*), P-cadherin (*CDH3*), occludin (*OCLN*), and connexin (*GJA1*) had a significant expression in expression compatible with EMT, while for desmoplakin (*DSP*), expression was increased [21]. On the contrary, the expression of genes associated with mesenchymal cells such as N-cadherin (*CDH2*), cyclin D1 (*CCND1*), fibronectin (*FN1*), vimentin (*VIM*), and transforming growth factor-beta 2 (*TGFB2*) [21] increased at 133 days in culture in comparison to their expression at 21 and 91 days (Table 3)*.* We also observed fibroblast-like shaped cells at 115 days in culture by optical microscopy (Fig. 2L). Moreover, *TRPM1* and *TRPM3* genes (transient receptor potential cation channel subfamily M members 1 and 3), essential for the maintenance of differentiation and blockage of EMT and coexpressed with the non-coding microRNAs 211 and 204, respectively, were downregulated after 133 days in culture [20].

**Table 3:** Expression of genes associated with EMT in the RPE cell cultures (21, 91, and 133 days of follow-up). Fold changes and statistical significance test were carried out following the DESeq2 method. ns: *q*-value > 0.05; *: *q*-value < 0.05; **: *q*-value < 0.01

		Average read count			Fold change (significance)		
Gene	Product	21 days	91 days	133 days	91 vs 21 days	133 vs 91 days	133 vs 21 days
*CDH1*	E-cadherin	5689	5314	819	1.00 (ns)	0.16 (*)	0.16 (*)
*CDH3*	P-cadherin	5416	5925	1328	1.17 (ns)	0.24 (**)	0.28 (**)
*DSP*	Desmoplakin	8078	7143	13,960	0.95 (ns)	1.97 (**)	2.06 (**)
*OCLN*	Occludin	248	143	77	0.62 (*)	0.36 (**)	0.54 (*)
*GJA1*	Connexin	9145	6269	2399	0.74 (**)	0.30 (**)	0.41 (**)
*CCND1*	Cyclin D1	31,387	37,586	72,308	1.29 (*)	2.03 (**)	2.63 (**)
*CDH2*	N-cadherin	6801	11,332	15,700	1.80 (**)	2.63 (**)	1.47 (*)
*FN1*	Fibronectin	62,444	206,629	590,157	3.59 (**)	10.79 (**)	3.01 (*)
*VIM*	Vimentin	132,109	146,674	284,423	1.20 (ns)	2.45 (**)	2.05 (**)
*TGFB2*	Transforming growth factor-beta 2	3364	4510	9749	1.45 (**)	2.28 (**)	3.31 (**)
*TRPM1*	Transient receptor potential cation channel subfamily M member 1	2915	2341	4	0.85 (ns)	0.00 (**)	0.00 (**)
*TRPM3*	Transient receptor potential cation channel subfamily M member 3	2396	1649	127	0.73 (ns)	0.08 (**)	0.06 (**)

Although once differentiated RPE cells maintain a static epithelial phenotype characterized by intercellular unions and apical-basal polarization, they can de-differentiate and acquire mesenchymal characteristics (non-polarization and migratory behavior) under certain conditions [20], including inflammation, oxidative stress, high glucose levels, and cellular senescence [22]. One of the typical hallmarks of EMT is the switch of E-cadherin adhesions for N-cadherin adhesions, but RPE cultures, including the ones from the present study, are characterized by a dominant expression of N-cadherin, implying an atypical regulation of EMT yet to understand [23]. However, from 91 to 133 days, a decrease in E-cadherin expression and an increase in N-cadherin was observed. Although GO enrichment analysis and expression of specific markers suggest the occurrence of EMT, these results should be additionally confirmed at the protein level and preferentially with a single-cell approach, since heterogeneity of cells in culture could mask analysis results.

### Multielemental level follow-up by ICP-MS during RPE degeneration

**Fig. 3:**
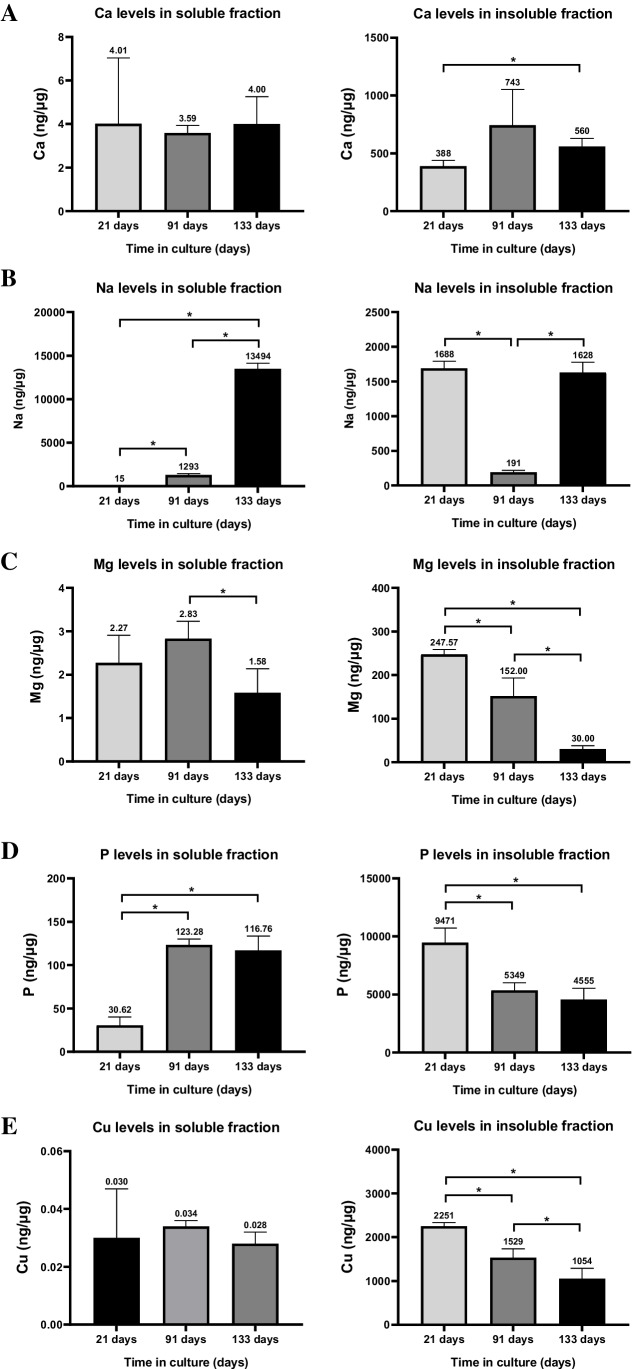
Levels of Ca (**A**), Na (**B**), Mg (**C**), P (**D**), and Cu (**E**) in the water-soluble and insoluble fractions of RPE cells at 21, 91, and 133 days in culture. Data is represented as concentration (ng·μg^−1^ total protein, in the case of the cytosol; and ng·g.^−1^ sample, in the case of the membranes) and error bars depict the standard deviation. *: significant *p*-value of two-way ANOVA (< 0.05)

Considering that the cell cultures showed phenotypic characteristics of degenerated RPE, we studied levels of Ca, Na, Mg, P, and Cu by FIA-ICP-MS (Fig. 3). Other elements such as Zn, Fe, Mn, and Se would be of interest in relation to RPE degeneration; however, for proper reliable analysis, we decided to focus on those listed above.

#### Calcium

Ca levels were determined in the water-soluble (cytosol) and insoluble fractions (membranes) of cell cultures by FIA-ICP-MS. Figure 3A compiles the mean values and standard deviation of Ca concentration ranging from 4.01 ± 3.02 ng·µg^−1^ (21 days) to 3.59 ± 0.34 ng·µg^−1^ (91 days) and 4.00 ± 1.24 ng·µg^−1^ (133 days) in the cytosol, and from 388 ± 50 ng·g^−1^ (21 days) to 743 ± 309 ng·g^−1^ (91 days) and 560 ± 329 ng·g^−1^ (133 days) in the membranous fraction. Slightly significant differences were only observed in the insoluble fraction when comparing 21 to 133 days, although seemed constant during RPE degeneration.

#### Sodium

Na levels in the water-soluble and insoluble fractions of cell cultures showed a striking upward tendency with time (Fig. 3B), suggesting metal dyshomeostasis. Na levels statistically significantly increased in the cytosol of RPE cells between 21 and 133 days in culture, from 15 ± 20 ng·µg^−1^ at 21 days to 1293 ± 145 ng·µg^−1^ at 91 days and to 13,495 ± 638 ng·µg^−1^ at 133 days, i.e., 86- and tenfold changes, respectively. However, Na levels within the insoluble fraction were similar at 21 (1688 ± 102 ng·g^−1^) and 133 (1628 ± 148 ng·g^−1^), showing a significant decrease at 91 days of culture (191 ± 26 ng·g^−1^). Furthermore, significant downregulation of several Na^+^/K^+^-ATPase coding genes from 21 to 133 days in culture (namely, *ATP1B1, ATP1B2*, *ATP1A1*, and *ATP1A3*) was observed, with the exception of the upregulation of *ATP1B3* (Table S3 of Supplementary Information). These changes suggest that a possible impairment of Na^+^/K^+^-ATPase could be related with the rise of Na inside dysfunctional RPE cells, although it is unknown at the protein level [24]. A downregulation of Na^+^/K^+^-ATPase (specifically, *ATP1B1*) was already described in RPE cells of DKO mice that showed loss of tight junctions mediated by ZO-1 and reduced TEER [25], similar to our 133-day-old RPE cell. Interestingly, some transcription factors associated with the expression of EMT-related genes, like TGF-β, have been described to downregulate the expression of Na^+^/K^+^-ATPase genes [26].

#### Magnesium

Mg levels (Fig. 3C) significantly decreased in the water-soluble fraction at 133 days (1.58 ± 0.56 ng·µg^−1^) when compared to 91 days (2.83 ± 0.40 ng·µg^−1^, 0.56-fold), showing no differences at 21 days. Similarly, a statistically significant decrease was observed in the membranous fraction from 21 days (247.57 ± 11.06 ng·g^−1^) to 91 days (152 ± 41 ng·g^−1^) and to 133 days in culture (30 ± 8 ng·g^−1^). Mg, the second most abundant intracellular cation, serves as a cofactor of multiple enzymes, particularly those involved in ATP synthesis, and its levels inside cells are tightly controlled but its dyshomeostasis can occur under pathological conditions [27]. Deficiency of Mg has been linked with multiple diseases and the improper homeostasis of other elements, since it also affects the Na^+^/K^+^-ATPase by depleting ATP sources, which in turn produces an increase in Na^+^ ions and a decrease in K^+^ ions inside the cells [28]. Additionally, deficiency of Mg can reduce Ca^2+^ export from the cell and increased cytosolic Na^+^ can lead to the release of cytosolic Ca^2+^ from the mitochondria [29]. The increase of Na levels observed in the cell cultures of 133 days could be related with a deficiency in ATP synthesis induced by Mg deficit, which would block Na^+^/K^+^-ATPase activity.

#### Phosphorus

The concentration of P (Fig. 3D) increased in the water-soluble fraction from 21 days (30.6 ± 9.5 ng·µg^−1^) to 91 (123.3 ± 6.6 ng·µg^−1^) and 133 days (116.8 ± 16.8 ng·µg^−1^) in culture (4.1-fold 91 vs 21 days; 3.8-fold 133 vs 21 days; *p*-value < 0.05), and deceased in an opposite way in the water-insoluble fraction from 21 days (9471 ± 1249 ng·µg^−1^) to 91 (5349 ± 666 ng·µg^−1^) and 133 days (4555 ± 985 ng·µg^−1^) cultured cells (0.6-fold 91 vs 21 days; 0.5-fold 133 vs 21 days; *p*-value < 0.05). This element is highly concentrated in the RPE mainly in the cell nuclei, cytoplasm, lipofuscin, and drusen [30]. It must be stressed that RPE cell cultures were quickly washed with DPBS before cell lysis. DPBS contains phosphate and therefore the levels of P in the water-soluble fraction could be affected by this buffer, although this would occur similarly in all cultures. Increased levels of P during the degeneration of RPE cells can be related to the enrichment of phosphorylation and protein modification processes found by gene expression analysis, which can indicate an altered proteostasis and increased formation of lipofuscin granules [31]. Interestingly, sub-RPE deposits contain hydroxyapatite spherules, i.e., calcium phosphate providing anchorage for proteins [32] and phospholipid enrichment contributing to the hyperfluorescent of hard drusen [33]. The observed increasing cytoplasmic levels of P during RPE degeneration may contribute to the abnormal formation of extracellular deposits.

#### Copper levels, copper transporters, and antioxidant enzymes requiring metals as cofactors

Cu levels in the cytosolic and membranous fractions of cell cultures are depicted in Fig. 3E. Cu levels in the water-soluble fraction varied from 0.030 ± 0.017 ng·µg^−1^ (21 days) to 0.034 ± 0.002 ng·µg^−1^ (91 days) and to 0.028 ± 0.004 ng·µg^−1^ (133 days), without statistically significant changes with time. In the membranous fraction, Cu levels varied from 2251 ± 79 ng·g^−1^ (21 days) to 1529 ± 207 ng·g^−1^ (91 days) and to 1054 ± 235 ng·g^−1^ (133 days), observing a significant decrease when comparing all studied times in culture (0.7-fold 91 vs 21, *p*-value < 0.05; 0.5-fold 133 vs 21, *p*-value < 0.05; 0.7-fold 133 vs 91 days, *p*-value < 0.05). Besides, Table 4 depicts the levels and gene expression changes of enzymatic antioxidants requiring metal as cofactors, i.e., superoxide dismutase (SOD), catalase (CAT), and glutathione peroxidase (GPX). During RPE cell culture degrading, there was a decrease in the expression levels of all SOD isoforms (SOD1, 2, and 3), CAT, GPX4, and GPX7 at 133 days in culture, while for GPX8, expression increased. Similarly, gene expression levels of mammalian Cu transporters are shown in Table 5, specifically the two homologous Cu-ATPases (ATP7A and ATP7B) responsible for decreasing Cu cytosolic concentration and the cellular importers CTR1 (high-affinity, codified by *SLC31A1* gene) and CTR2 (low affinity, codified by *SLC31A2* gene). After 91 days, RPE cultured cells increased the expression of CTR2 importer and decreased the ATB7B exporter, being more dramatically at 133 days.

**Table 4:** Temporal changes in the gene expression of antioxidant enzymes requiring metals as cofactors, in RPE cells at 21, 91, and 133 days in culture. Fold changes and statistical significance test were carried out following the DESeq2 method. ns: *q*-value > 0.05; *: *q*-value < 0.05; **: *q*-value < 0.01

	Average read count			Fold change (significance)		
Gene	21 days	91 days	133 days	91 vs 21 days	133 vs 91 days	133 vs 21 days
*SOD1*	2359	1562	1537	0.71 (**)	1.05 (ns)	0.74 (**)
*SOD2*	2000	1708	1231	0.91 (ns)	0.77 (ns)	0.70 (**)
*SOD3*	592	734	208	1.32 (ns)	0.30 (ns)	0.40 (**)
*CAT*	717	600	387	0.90 (ns)	0.69 (ns)	0.62 (**)
*GPX1*	6540	7423	5426	1.22 (*)	0.78 (*)	0.95 (ns)
*GPX3*	2243	3302	2201	1.58 (**)	0.71 (ns)	1.12 (ns)
*GPX4*	5012	5086	2689	1.09 (ns)	0.56 (**)	0.61 (**)
*GPX7*	1119	1038	549	1.00 (ns)	0.56 (**)	0.56 (**)
*GPX8*	2105	3175	3023	1.63 (**)	1.01 (ns)	1.64 (**)

**Table 5:** Temporal changes in the gene expression of copper transporters in RPE cells at 21, 91, and 133 days in culture. Fold changes and statistical significance test were carried out following the DESeq2 method. ns: *q*-value > 0.05; *: *q*-value < 0.05; **: *q*-value < 0.01

	Average read count			Fold change (significance)		
Gene	21 days	91 days	133 days	91 vs 21 days	133 vs 91 days	133 vs 21 days
*SLC31A1*	1168	1149	1040	1.06 (ns)	0.96 (ns)	1.02 (ns)
*SLC31A2*	106	150	624	1.52 (*)	4.40 (**)	6.70 (**)
*ATP7A*	318	278	215	0.94 (ns)	0.82 (ns)	0.77 (ns)
*ATP7B*	512	315	202	0.66 (**)	0.68 (*)	0.45 (**)

Cu levels remained constant in the cytosolic fraction of RPE cells along their progressive degeneration, while the concentration in the cellular membranes significantly decreased at 91 and 133 days, similar to the observed downregulation of SOD, responsible for Cu homeostasis [34] and changes in Cu transporters that may try to regulate intracellular levels of the element. This downregulation of SOD may contribute to improper intracellular Cu control, probably triggering the accumulation of Cu in the choroid-RPE tissue of cadaveric AMD cases [35]. The observed altered metal and metalloprotein homeostasis may be associated with the impairment of the antioxidant defenses, the increase of oxidative stress, and the risk of RPE degeneration during ageing [36], being compromised proper Cu muffling exacerbated during cell culture ageing. We therefore examined changes in antioxidant enzymes using metal as cofactors. RPE in vitro degeneration contributed to the decrease of the expression levels of specific isoforms of antioxidant enzymes SOD, CAT, and GPX [36]. Downregulation of these enzymes, specifically those requiring Cu, indicates decreased antioxidant capacity of RPE cells during their degeneration and probably lower tolerance to oxidative stress. For example, the decrease in the CAT gene expression at 133 days of degenerating RPE cells relates to the observed reduction in CAT activity during ageing and AMD [37]. Similarly, mice deficient in SOD that uses Cu as a cofactor for superoxide anion dismutation showed elevated levels of oxidative species and develop an AMD-like phenotype [38], while knockdown of SOD produced pathological lesions similar to those observed in “dry” AMD [39].

## Conclusions

FIA-ICP-MS analysis along with gene expression and immunohistochemistry assays has been explored to study trace element changes in cell biological samples from an in vitro model of AMD. As a proof of concept, this study revealed a possible role of essential elements during progressive degeneration of the RPE, showing the importance of elemental MS in cell biology. Four-month-old cultures of RPE showed both differentiated and well-functioning cells along with imbalanced apoptotic processes, cells undergoing EMT, and evident disequilibrium in metal homeostasis owing to dysregulation of metal muffling proteins, transporters, antioxidant enzymes, and metal levels. However, the evident heterogeneity of cell populations along cell cultures, and most prominently at the end of the degeneration of RPE cells, should be highlighted when interpreting data and calls for further studies using a single-cell approach like SC ICP-ToF–MS or space-resolved MS to give a clearer insight into the mechanism of cell degeneration, the physiological time dependence, and the possible role of metals.

### Supplementary Information

Below is the link to the electronic supplementary material.

Supplementary file1 (DOCX 310 KB)
