# Supplementary material for: Elemental mass spectrometry to study metallo-transcriptomic changes during the in vitro degeneration of the retinal pigment epithelium
Source: Anal Bioanal Chem. 2023 Jul 29;416(11):2699–710. doi: 10.1007/s00216-023-04880-8 (PMC11009741; doi:10.1007/s00216-023-04880-8)
Supplement: Supplementary file 1 — Supplementary file1 (DOCX 310 KB) [file 216_2023_4880_MOESM1_ESM.docx]

Supplementary Information

# Elemental mass spectrometry to study metallo-transcriptomic changes during the *in vitro* degeneration of the retinal pigment epithelium

Ana Álvarez-Barrios^1,2^, Lydia Álvarez^1,3*^, Rosario Pereiro^2^, Héctor González-Iglesias^4*^

^1^ Fundación de Investigación Oftalmológica, Avda. Dres. Fernández-Vega. 34, 33012 Oviedo, Spain

^2^ Department of Physical and Analytical Chemistry, University of Oviedo, Julián Clavería, 8, 33006 Oviedo, Spain

^3^ Instituto Oftalmológico Fernández-Vega, Avda. Dres. Fernández-Vega, 34, 33012 Oviedo, Spain

^4^ Instituto de Productos Lácteos de Asturias, Consejo Superior de Investigaciones Científicas (IPLA-CSIC), Villaviciosa, Spain

*Corresponding authors: Héctor González-Iglesias ([hectorgi@ipla.csic.es](mailto:hectorgi@ipla.csic.es)); Lydia Álvarez ([l.alvarez@fio.as](mailto:l.alvarez@fio.as)).

**Supplementary Material Description:** The supplementary material contains supplementary data and information of the Results section.

**RESULTS**

**Barrier function**

***Transepithelial electrical resistance***

The transepithelial electrical resistance (TEER) has been monitored during the evolution of RPE cell cultures, according to Figure S1.

####

**Figure S1.** Temporal evolution of TEER in cell cultures. Data points represent the mean normalized TEER values (expressed in %) and the 95% CI, depicted as error bars.

**Specific time-associated changes in the transcriptome**

***Gene Ontology (GO) enrichment analysis***

Differentially expressed genes (DEGs) among time points of culture cells were identified and aligned to the Gene Ontology (GO) database for biological processes enrichment analysis. The top 20 most significantly enriched processes were selected and depicted as bubble charts in Figure S2.

Figure S2. Bubble chart of the 20 most significantly enriched biological processes (GO database) between 21 and 133 days in culture. Each process (in the y-axis) is depicted by a bubble, its size represents the number of DEGs annotated to a GO term, and its color shows the enriched significance (Q-value). X-axis represents the enrichment ratio of each process (ratio of the number of enriched genes annotated to the GO term in relation to the total number of genes annotated to that GO term in the species).

**Multielemental levels in the culture media by ICP-MS**

Analytical parameters.

**Table S1**. Analytical parameters for FIA-ICP-MS analysis of Na, Mg, P, Ca and Cu. LODs (Limits of Detection) were calculated considering the calibration curve for each analyte as the ratio of three times the standard deviation of the intercept and the calibration slope. LOQs (Limits of Quantification) were calculated considering c the calibration curve for each analyte as the ratio of ten times the standard deviation of the intercept and the calibration slope.

| **Element** | **LOD (ppb)** | **LOQ (ppb)** | **Sensitivity (ppb)** | **Intercept** | **R^2^** |
| --- | --- | --- | --- | --- | --- |
|  |  |  |  |  |  |
| Ca | 11 | 36 | 0.0003 | 0.0209 | 0.9999 |
| Na | 43 | 142 | 0.0165 | 0.071 | 0.9999 |
| Mg | 43 | 144 | 0.0048 | 0.0369 | 0.9991 |
| P | 155 | 516 | 0.0001 | 0.0031 | 0.9999 |
| Cu | 1 | 3 | 0.0768 | 0.0488 | 0.9993 |

Multielemental quantification of Ca, Na, Mg, P and Cu was carried out in the culture medium of RPE cells by FIA-ICP-MS.

**Table S2.** Averaged concentration of Ca, Na, Mg, P and Cu in cell media during the follow-up of RPE cells, i.e., without cells and with RPE cells at 21, 91 and 133 days in culture. Data is showed as averaged concentration (ppm or ppb) and standard deviation.

| **Element** | **Cell media** | **Days** | | |
| --- | --- | --- | --- | --- |
|  |  | **21** | **91** | **133** |
| Ca (ppm) | 56±6 | 58±25 | 51±20 | 55±4 |
| Na (ppm) | 2647 ±110 | 2461 ±400 | 2312 ±537 | 2388 ±111 |
| Mg (ppb) | 7955±827 | 7667 ±500 | 7497 ±796 | 6672±911 |
| P (ppm) | 35±5 | 39±14 | 37±8 | 29±2 |
| Cu (ppb) | 8±2 | 10±7 | 8±2 | 6±1 |

**Sodium homeostasis: Na^+^/K^+^-ATPase**

Expression of Na^+^/K^+^-ATPase coding genes in primary RPE cells throughout 21-133 days was studied.

**Table S3.** Expression of Na^+^/K^+^-ATPase genes at 21, 91 and 133 days in culture. Fold-Changes and statistical significance test were carried out following the DESeq2 method. ns: q-value > 0.05; *: q-value < 0.05; **: q-value < 0.01

|  | **Average Read Count** | | | **Fold-Change (Significance)** | | |
| --- | --- | --- | --- | --- | --- | --- |
| **Gene** | **21 days** | **91 days** | **133 days** | **91 vs 21 days** | **133 vs 91 days** | **133 vs 21 days** |
| *ATP1B1* | 5091 | 2782 | 1400 | 0.59 (**) | 0.54 (*) | 0.31 (**) |
| *ATP1B2* | 343 | 386 | 24 | 1.20 (ns) | 0.07 (*) | 0.08 (**) |
| *ATP1B3* | 1690 | 2525 | 2738 | 1.61 (**) | 1.15 (ns) | 1.85 (**) |
| *ATP1B4* | 5 | 5 | 2 | 1.14 (ns) | 0.30 (ns) | 0.34 (ns) |
| *ATP1A1* | 10197 | 6699 | 6420 | 0.71 (**) | 1.02 (ns) | 0.72 (**) |
| *ATP1A2* | 2 | 3 | 2 | 1.72 (ns) | 0.80 (ns) | 1.37 (ns) |
| *ATP1A3* | 3834 | 625 | 59 | 0.17 (**) | 0.10 (**) | 0.02 (**) |
